# Anthocyanin Genes Involved in the Flower Coloration Mechanisms of *Cymbidium kanran*

**DOI:** 10.3389/fpls.2021.737815

**Published:** 2021-10-12

**Authors:** Zhuang Zhou, Zhen Ying, Zhigang Wu, Yanping Yang, Shuangbin Fu, Wan Xu, Lijuan Yao, Aiping Zeng, Jian Huang, Siren Lan, Xiaole Wang, Zhongjian Liu

**Affiliations:** ^1^Zhejiang Institute of Subtropical Crops, Zhejiang Academy of Agricultural Sciences, Wenzhou, China; ^2^Key Laboratory of National Forestry and Grassland Administration for Orchid Conservation and Utilization at College of Landscape Architecture, Fujian Agriculture and Forestry University, Fuzhou, China

**Keywords:** orchids, *Cymbidium kanran*, anthocyanins, *CkCHS-1*, *CkDFR*, *CkANS*, flower coloration

## Abstract

The Orchidaceae, otherwise known as orchids, is one of the largest plant families and is renowned for its spectacular flowers and ecological adaptations. Various polymorphisms of orchid flower colour can attract pollinators and be recognised as valuable horticultural ornamentals. As one of the longest historic cultured orchids, *Cymbidium kanran* has been domesticated for more than 2,500 years and is an ideal species to study coloration mechanisms because of plentiful variations in floral coloration and abundant traditional varieties. In this study, we used two distinct colour-type flowers of *C. kanran* as experimental materials to elucidate the mechanism of flower coloration. High-performance liquid chromatography (HPLC) analysis revealed that anthocyanins in purple-red-type flowers include three types of anthocyanidin aglycones, peonidin, malvidin, and cyanidin, whereas anthocyanins are lacking in white-type flowers. Through comparative transcriptome sequencing, 102 candidate genes were identified as putative homologues of colour-related genes. Based on comprehensive correlation analysis between colour-related compounds and gene expression profiles, four candidates from 102 captured genes showed a positive correlation with anthocyanidin biosynthesis. Furthermore, transient expression of *CkCHS-1, CkDFR*, and *CkANS* by particle bombardment confirmed that recovery of their expression completed the anthocyanin pathway and produced anthocyanin compounds in white-type flowers. Collectively, this study provided a comprehensive transcriptomic dataset for *Cymbidium*, which significantly facilitate our understanding of the molecular mechanisms of regulating floral pigment accumulation in orchids.

## Introduction

Orchids are important ornamental plants characterised by their fantastic floral morphology and high levels of corolla-colour polymorphism and variability, which have played an important role in pollination and have excited biologists since Darwin (Gigord et al., 2001; Aguiar et al., 2020; Basist et al., 2021). As an important member of orchids, *Cymbidium* species are probably the earliest orchid, having been cultivated since before the time of Confucius (551–479 BC) for traditional use in worship and ornamentation (Chen and Tang, 1982; Du Puy et al., 2007). Currently, members of *Cymbidium* are still very prevalent and dominate the world floriculture markets (Singh et al., 2019). Although the mechanism of genes encoding enzymes for flower coloration in orchids has been studied in some species (Mudalige-Jayawickrama et al., 2005; Han et al., 2006; Hieber et al., 2006; Chen et al., 2011; Hsiao et al., 2011; Liu et al., 2012; Yu et al., 2018), as one of the longest historic floricultural plants, *Cymbidium kanran* attracts wide attention. However, its coloration mechanism remains unclear.

Anthocyanins, as a series of secondary metabolites, are water-soluble pigments that contribute colours from orange, pink, red, magenta, purple, blue, cyan to “black” (Hatier and Gould, 2007; Tanaka et al., 2008; Petroni and Tonelli, 2011; Moreau et al., 2012; Li et al., 2014). These pigments are an ideal model for genetics, molecular biology, and cell biology (Passeri et al., 2016). To date, hundreds of anthocyanins have been extensively studied and characterised, which were originally based on six common types of anthocyanidins (chromophores of anthocyanins), namely, petunidin, pelargonidin, delphinidin, cyanidin, malvidin, and peonidin. Accordingly, the synthetic route of anthocyanins and important related genes are well-studied and characterised in some model and non-model plants. Anthocyanins are derived from flavonoid biosynthetic pathways, and their biosynthesis has been divided into three stages (Grotewold, 2006). The first stage is phenylalanine and phenylpropanoid metabolism, which is shared among other secondary metabolisms. The second stage, flavonoid metabolism, is the crux of anthocyanin biosynthesis. Chalcone synthase (CHS) is the first key enzyme to produce chalcones, the precursor for all classes of flavonoids. Subsequently, an enzymatic reaction of chalcones to naringenins is catalysed by chalcone isomerase (CHI), which is followed by a conversion to dihydrokaempferol (DHK) by flavanone 3-hydroxylase (F3H). The last step involves two participants, flavonoid 3'-hydroxylase (F3'H) and flavonoid 3', 5'hydroxylase (F3' 5'H), corresponding to the respective products dihydroquercetin (DHQ) and dihydromyricetin (DHM). The final stage is anthocyanin metabolism, including two rate-limiting steps catalysed by dihydroflavonol reductase (DFR) and anthocyanidin synthase (ANS). Three kinds of products, delphinidin, pelargonidin, and cyanidin, undergo several modifications, such as glycosylation or methylation, by UDP-glucoside: flavonoid 3-O-glucosyltransferase (3GT) and anthocyanin methyltransferase (AMT).

Transgenic technology, expression, and suppression of the level of the anthocyanin synthetic gene have been verified as feasible to study the accumulation mechanism of anthocyanin in many plants. With the expression of *UFGT*, the skin colours of the *Vitis* and *Litchi* were turned red or violet from green (Ramazzotti et al., 2008; Zhao et al., 2012). Overexpression of *SmANS* from *Salvia miltiorrhiza* enhanced anthocyanin accumulation in the petals of *S. miltiorrhiza* f. *alba* and resulted in purple-red colour from white colour (Li et al., 2019). Using RNA interference (RNAi) technology to suppress *CHS, ANS*, and *F3'5'H*, the petals of gentian plants exhibited pure white to pale-blue and magenta flowers accordingly (Nakatsuka et al., 2008). Consequently, using transgenic approaches to introduce exogenous anthocyanin genes into the targeted plant can obtain the desired coloration traits to produce new flower colour (Meyer et al., 1987; Katsumoto et al., 2007).

**Figure 1:**
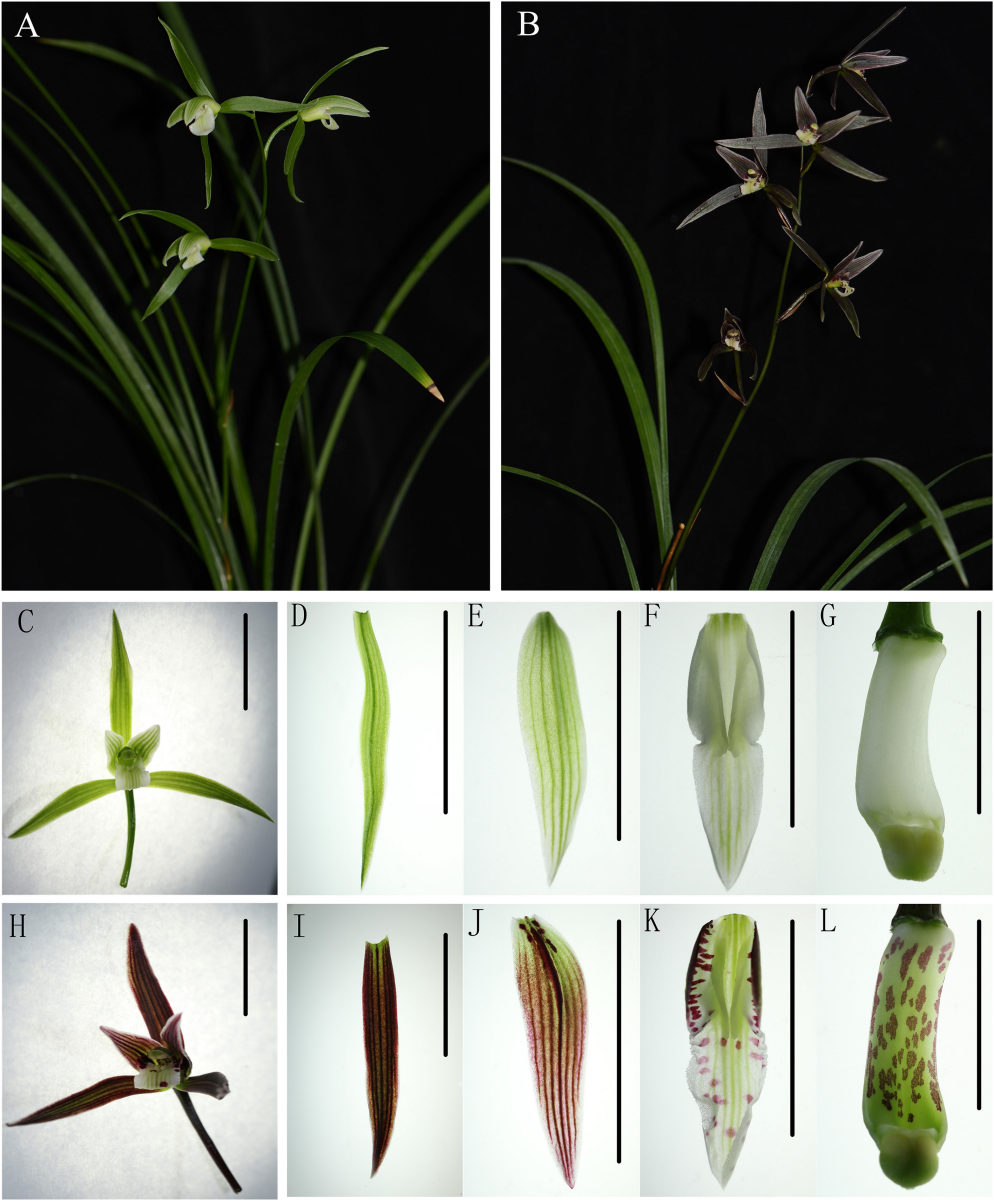
The phenotype of purple-red and white *Cymbidium kanran*. **(A)** The plant of purple-red *C. kanran*. **(B)** The plant of white *C. kanran*. **(C–G)** Flower, sepal, petal, lip, and gynostemium of white *C. kanran*. **(H–L)** Flower, sepal, petal, lip, and gynostemium of purple-red *C. kanran*. Scale bar = 1 cm.

*Cymbidium kanran* is an ideal species to study coloration mechanisms because of numerous variations in floral coloration and abundant traditional varieties. In this study, we used purple-red flowers (with many purple-red pigments) and white flowers (greenish-white without purple-red pigments) as research materials (Figure 1). Through a combination of transcriptome sequencing, chemical, and molecular analysis, the mechanism of anthocyanin deficiency in white was preliminarily revealed. This dataset provided a novel resource to study and to understand the molecular mechanisms of coloration in *C. kanran*.

## Materials and Methods

### Plant Material and Freehand Sectioning

White-type flowers and purple-red-type flowers of *C. kanran* were used as research materials in this study and cultivated in the greenhouse of Zhejiang Institute of Subtropical Crops in Wenzhou City, China (120°37′53′′E, 28°0′8′′N). For the first one, it has white flowers (Figure 1A), for the second, it has purple-red flowers with purple-red sepals/petals and white lip with purple-red spots (Figure 1B). The buds were about 2, 3 cm, 1–2 days before anthesis, and opened flowers were used for Real-Time PCR analysis. At the same time, about 3 cm length buds were selected for transcriptome sequencing and fully opened flowers were selected for freehand sectioning and pigment analysis. When harvested, all the materials were immediately frozen in liquid nitrogen (LN) and stored at −80°C except the freehand sectioning samples. Petals or lips of fully opened flowers were perpendicularly cut quickly to their longitudinal axis by hand sectioning with a new sharp double-edged razor blade. The sections were immersed in water and observed under an optical microscope.

### RNA Extraction, Library Construction, and RNA-seq

The total RNA of each sample was isolated using TRIzol Reagent (Invitrogen Life Technologies, Carlsbad, CA, USA). Then, the integrity and concentration were checked on a 1% agarose gel and a NanoDrop ND-1000 spectrophotometer (NanoDrop Technologies, Wilmington, DE, USA). The enriched mRNA was broken into short fragments, which were used to synthesise first- and second-strand cDNA. After that, the construction of the cDNA library was performed following the instructions of the manufacturer of the NEBNext Ultra RNA Library Prep Kit for Illumina (NEB, E7530, New England Biolabs, Ipswich, MA, USA) and NEBNext Multiplex Oligos for Illumina (NEB, E7500). Finally, the libraries of *C. kanran* buds were sequenced using an Illumina HiSeq™ 2500 by Biomarker Biotechnology Corporation (Beijing, China).

### RNA-seq Data Assembly, Annotation, and Differential Expression Analysis

Raw data obtained by RNA-seq were primarily processed through in-house Perl scripts. In this step, clean reads were acquired by removing reads containing adapters, reads containing poly-N and low-quality reads from raw data. In addition, the Q20, Q30, GC content, and sequence duplication levels of the clean data were calculated to evaluate the sequencing quality. All the subsequent analyses were based on clean data with high quality. Transcriptome assembly was accomplished based on the fq file using Trinity software (https://github.com/trinityrnaseq/trinityrnaseq/wiki) (Grabherr et al., 2011) with min_kmer_cov set to two by default and all other parameters set to default.

Gene function was annotated by BLASTX (Altschul et al., 1997) program (http://www.ncbi.nlm.nih.gov/) based on the following databases: Nr (NCBI non redundant protein sequences); Nt (NCBI non-redundant nucleotide sequences); Pfam (Protein family); KOG/COG (Clusters of Orthologous Groups of proteins); Swiss-Prot (a manually annotated and reviewed protein sequence database); KO (KEGG Orthologue database); and GO (Gene Ontology).

Gene expression levels were estimated using RSEM software (http://deweylab.github.io/RSEM/) (Li and Dewey, 2011) for each sample. Differential expression analysis of the two samples was performed using DESeq (http://www.bioconductor.org/packages/3.8/bioc/html/DESeq.html). The resulting *P*-values were adjusted with the Benjamini-Hochberg method to control the false discovery rate. Genes with an adjusted *P* < 0.05 found by DESeq were identified as differentially expressed.

### Gene Validation and Expression Analysis

Total RNA was isolated from *C. kanran* buds and flowers using the ZR Plant RNA MiniPrep Kit (Zymo Research, Irvine, CA, USA), and 1 μg RNA was used to synthesise oligo(dT)-primed first-strand cDNA by the QuantiTect Reverse Transcription Kit (QIAGEN, Hilden, Germany) following the protocol of the manufacturer. The qRT-PCRs were performed in a 7300 Real-Time PCR System (Applied Biosystems, Foster Arthritis 5 City, CA, USA) using an SYBR premix Ex TaqTM Kit (TaKaRa, Kyoto, Japan). The reference gene we chose was actin (c114989.graph_c0). We calculated the relative expression level by the 2-^Δ^^Δ^CT method (Livak and Schmittgen, 2001).

### Measurement of Flower Flavonoids

We used the relative quantitative method to analyze flavonoids from white and purple-red *C. kanran* opened flowers. Approximately 0.1 g (fresh weight) was extracted in 1 ml 70% methanol at 4°C for 1 day. After centrifugation for 5 min at 12,000 rpm, the supernatant was collected. Before analysis by high-performance liquid chromatography (HPLC), the extract was filtered to remove impurities. Samples were analysed using LC-ESI-QTRAP-MS/MS with an Agilent 1200 Series HPLC system (Agilent Technologies, Waldbronn, Germany) coupled to an Applied Biosystems 4000 QTRAP (Darmstadt, Germany), and quantification of flavonoids was carried out in MRM mode. The analytical conditions were as described previously (Chen et al., 2013). Data acquisition, peak integration, and calculations were performed using Analyst 1.5 software (AB Sciex, MA, USA). We quantified the anthocyanin and flavonol contents of white, purple-red, and intermediate phenotype *C. kanran* flowers for Pearson's correlation coefficient in a different way. Approximately, 0.2 g (fresh weight) was extracted in 1 ml methanol: acetic acid: water (80:2:18, by vol.) at 4°C for 1 day. After centrifugation for 5 min at 12,000 rpm, the supernatant was collected, and the pellet was re-extracted for the second time. The combined supernatant was hydrolyzed in boiling water for 1 h and then quantitated to 2 ml. Before analysis by HPLC, the extract was filtered to remove impurities. HPLC was performed as previously described (Wang et al., 2014). Myricetin, kaempferol, quercetin, peonidin, malvidin, delphinidin, pelargonidin, and cyanidin (Sigma-Aldrich, China) were used for qualitative and quantitative analysis. Mean values and SDs were obtained from three replicates.

### Construction for Transient Expression by Particle Bombardment

According to the gene transcript level and flavonoid content, *CkCHS-1, CkDFR*, and *CkANS* were screened as key genes encoding enzymes in the flavonoid metabolic pathway of *C. kanran*. Therefore, the three genes were divided into three groups, single genes, double genes, and triple genes, to be validated for transient expression by particle bombardment. The genes driven individually by the 35S promoter were cloned into the pCAMBIA1305.1 vector at the NcoI restriction site. The recombinant plasmid DNA was extracted and purified using an EndoFree Plasmid Giga Kit (QIAGEN, Hilden, Germany). Before bombardment, healthy white-type *C. kanran* flowers were collected, and the following procedure was conducted as described in the study by Ma et al. (2008).

## Results

### Phenotypic Characterisation

**Figure 2:**
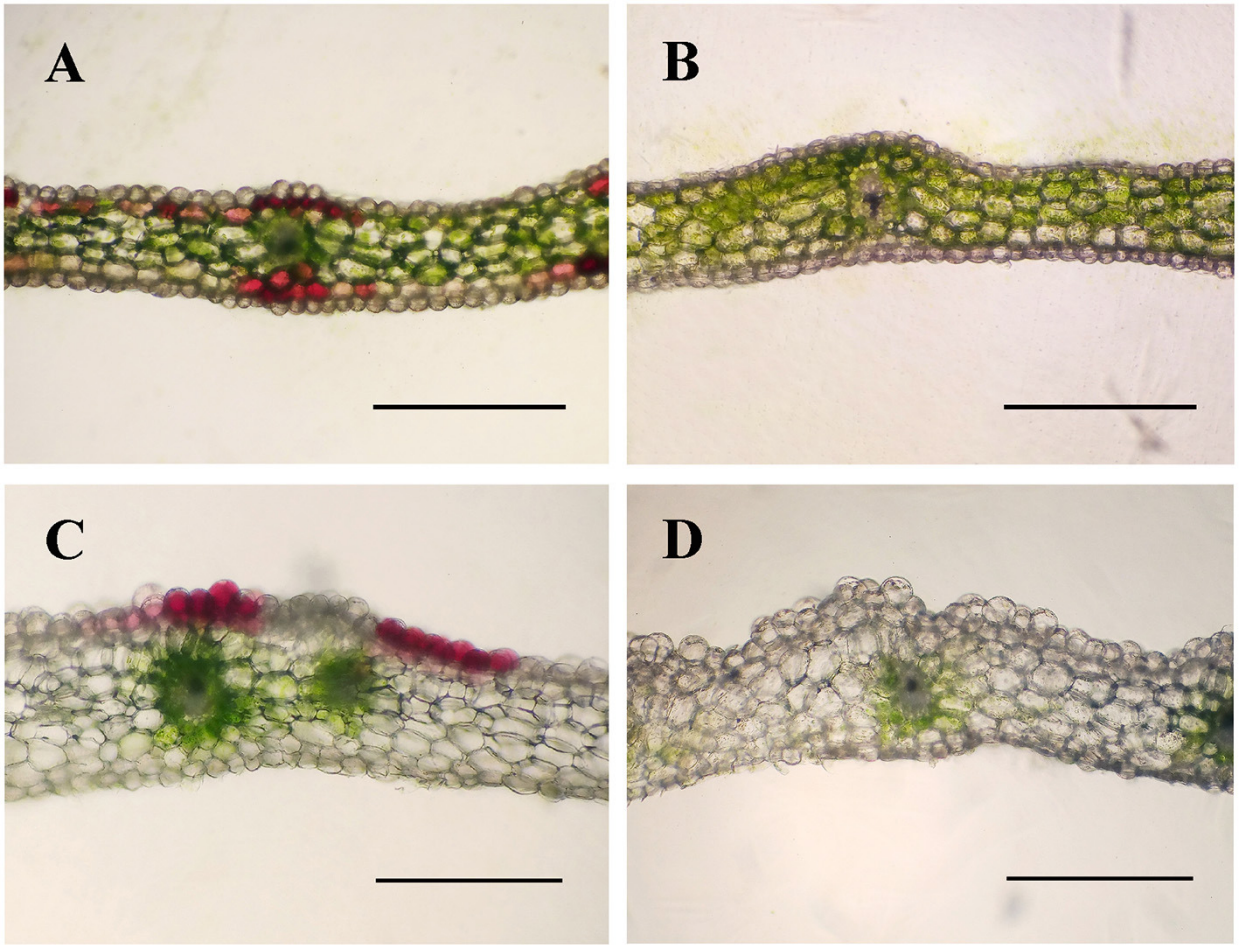
Cross-sections of purple-red and white *C. kanran* showing the different colour patterns in petals and lips. Anatomic structure of purple-red *C. kanran* petals **(A)** and lips **(C)**, white *C. kanran* petals **(B)**, and lips **(D)**. Scale bar = 500 μm.

In the process of domestication and cultivation of *C. kanran*, it is important to note the colour variety of the flowers. The most well-known cultivar, white-type *C. kanran*, has greenish-white petals and a white lip, both with greenish stripes (Figures 1A,C–G). Compared with white-type flowers, purple-red flowers exhibited purple-red petals with stripes of the same colour and a white lip with red spots and greenish stripes (Figures 1B,H–L). Because of the striking contrast in flower colour, white and purple-red *C. kanran* are the optimal candidates to reveal the flower coloration mechanism. To study the distribution of anthocyanin production in perianth cells, we analysed petal and lip cross-sections from white and purple-red *C. kanran* (Figure 2). In the petals, anthocyanins accumulated in the region from subepidermal cells to the xylem (Figure 2A), while in the lip, anthocyanins accumulated only in the epidermal cells of the adaxial surface (Figure 2C). However, white flowers showed no purple-red pigments (anthocyanins) in cells except chlorophyll (Figures 2B,D).

### Flavonoid Content

**Figure 3:**
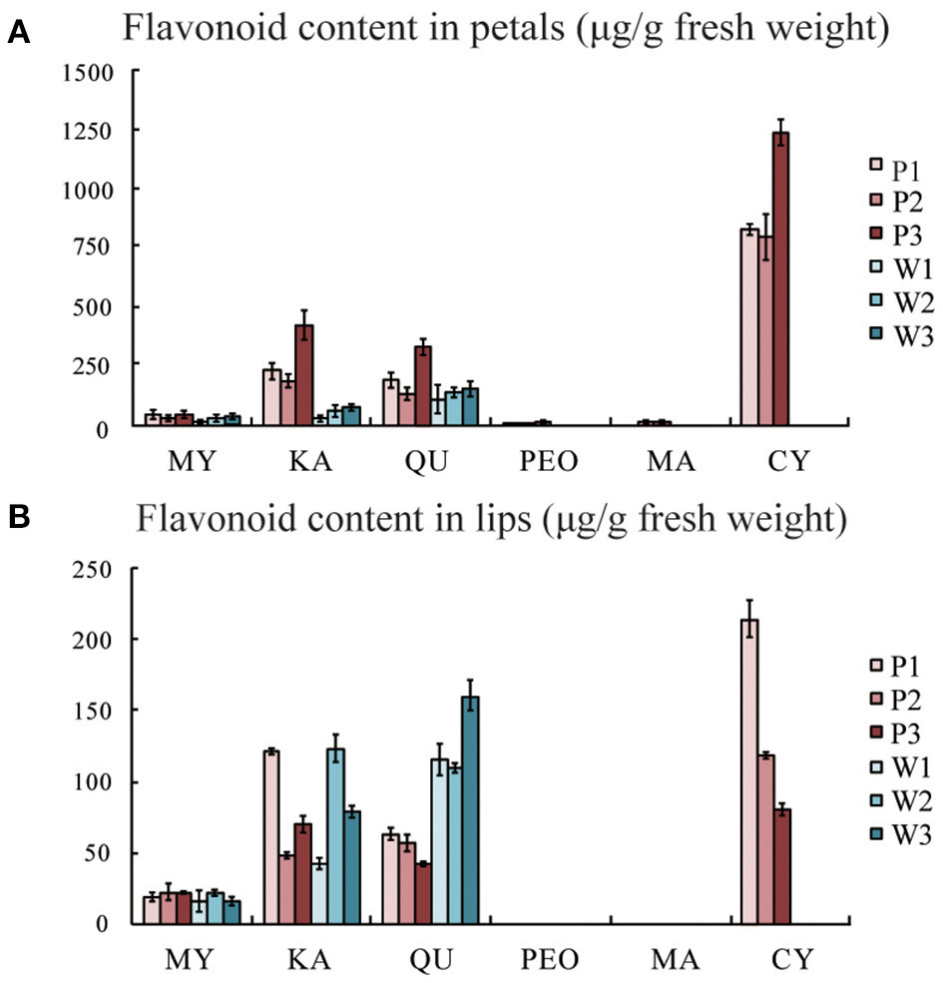
Flavonoid composition analysis by HPLC from flowers of purple-red and white *C. kanran*. **(A)** Flavonoid content in petals. **(B)** Flavonoid content in lips. MY, myricetin; KA, kaempferol; QU, quercetin; PEO, peonidin; MA, malvidin; CY, cyanidin; P1, purple-red *C. kanran* 1; W1, white *C. kanran* 1.

Anthocyanins, as the largest group of water-soluble pigments, are responsible for most of the purple, blue, and red colours in the plants. In this study, we quantified the flavonoids of purple-red and white *C. kanran* flowers by a relative quantitative method. In purple-red flowers, only cyanidin, peonidin, and malvidin were present, and the cyanidin content was far greater than that of peonidin and malvidin. Delphinidin and pelargonidin-type anthocyanins were not detected, which probably was due to their extremely low content. Interestingly, the contents of myricetin (MY), kaempferol (KA), and quercetin (QU) in the white petals were lower than those in the purple-red *C. kanran* petals. However, it is worth noting that QU was detected in the white lip at a concentration two times higher than that in the purple-red lip, while the concentrations of MY and KA were similar in both study objects (Figure 3).

### RNA-seq, Assembly, and Differential Expression Analysis

To illustrate the mechanism underlying the variation in flower coloration and to identify potential genes involved in this process, the petals and lips of purple-red and white *C. kanran* were used to generate four libraries for high-throughput sequencing. After quality cheques, 61.90 Gb clean data were obtained, and the Q30 percentages (percentage of sequences with sequencing error rates <0.1%) of every sample were higher than 87.72%. These data showed that the sequencing quality and throughput were reliable enough to carry out further research. Short reads were then assembled into 181,335 transcripts and 74,713 unigenes by Trinity with mean lengths of 1,230 bp and 887 bp, respectively. After searching the reference sequences using BLASTX against KEGG, COG, KOG, GO, nr, and Swiss-Prot, we obtained a total of 26,930 unigenes providing a significant BLAST result.

**Figure 4:**
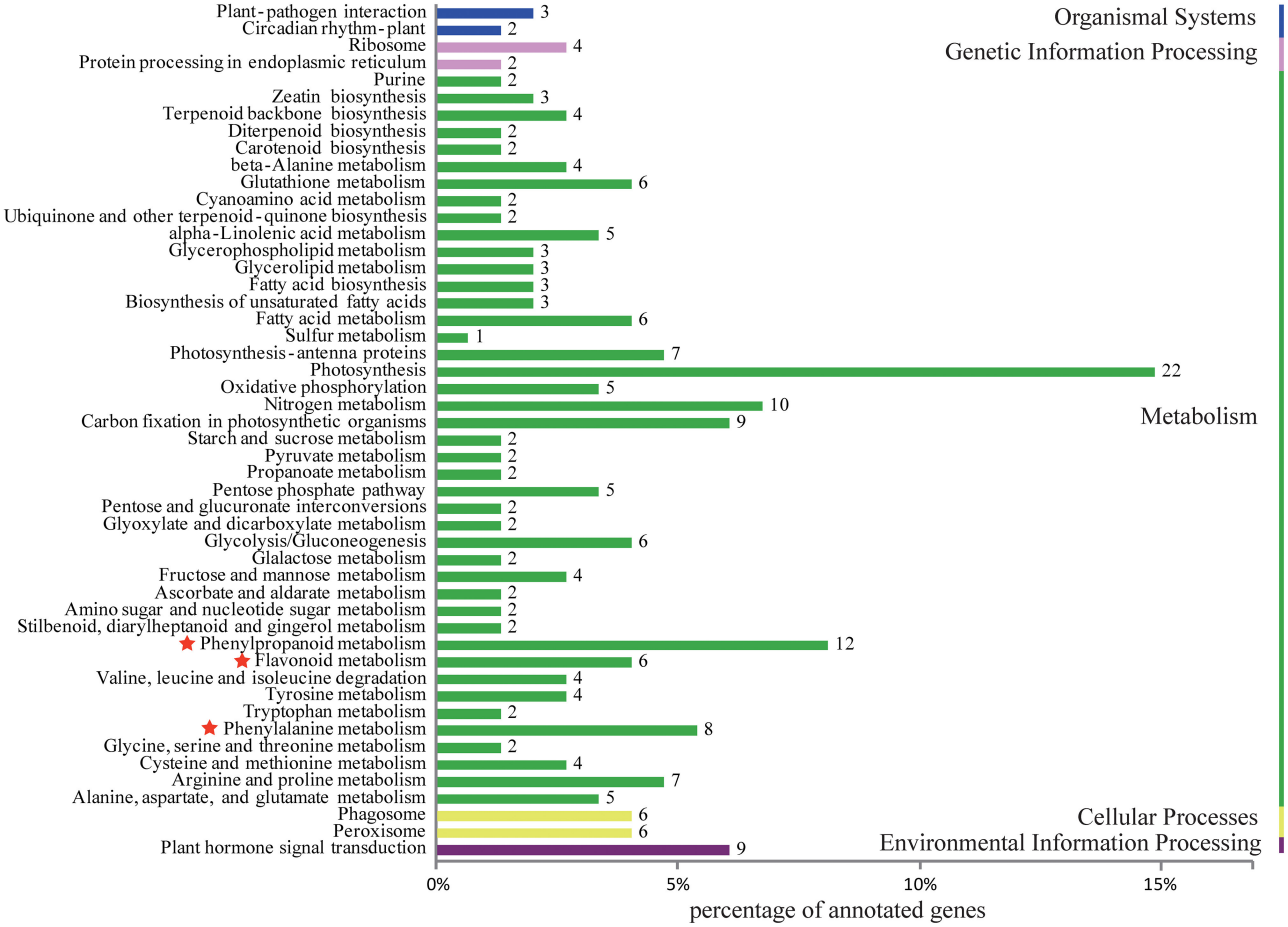
Histogram of KEGG classification for different colours of *C. kanran*. Functional classification of differentially expressed unigenes, considering both purple-red and white *C. kanran*. Classification followed the KEGG annotations. The red star shows the category we focused on.

Differentially expressed unigenes were grouped into 50 functional categories following KEGG annotation (Figure 4). The most prevalent functional category was photosynthesis, followed by phenylpropanoid metabolism, which was located upstream of flavonoid metabolism. Members involved in phenylalanine and flavonoid metabolism were also major parts of differentially expressed unigenes. These results indicated that the alternation of flavonoid metabolism was the underlying factor leading to a striking contrast in flower colour between purple-red and white *C. kanran*. Furthermore, the crucial genes taking part in three secondary metabolic pathways (phenylpropanoid, flavonoid, and anthocyanin metabolism referring to KEGG pathways) are listed in Table 1, consisting of 102 unigenes.

**Table 1:** Candidate genes related to flower pigmentation of *Cymbidium kanran*.

**Function**	**Name**	**Target description**	**KO id (EC No.)**	**No. All^**a**^**
Phenylpropanoid biosynthesis	C4H	Trans-cinnamate 4-monooxygenase	K00487 (1.14.13.11)	4
	C3H	Coumaroylquinate 3′-monooxygenase	K09754 (1.14.13.36)	1
	CCoAOMT	Caffeoyl-CoAO-methyltransferase	K00588 (2.1.1.104)	7
	HCT	Shikimate hydroxycinnamoyl transferase	K13065 (2.3.1.133)	4
Flavonoid biosynthesis	CHS	Chalcone synthase	K00660 (2.3.1.74)	14
	CHI	Chalcone isomerase	K01859 (5.5.1.6)	2
	F3H	Flavanone 3-hydroxylase	K00475(1.14.11.9)	10
	F3′H	Flavonoid 3′-hydroxylase	K05280 (1.14.13.21)	9
	F3′5′H	Flavonoid 3′5′-hydroxylase	K13083 (1.14.13.88)	1
	DFR	Dihydroflavonol-4-reductase	K13082 (1.1.1.219)	3
	ANS	Anthocyanidin synthase	K05277 (1.14.11.19)	6
	ANR	Anthocyanidin reductase	K08695 (1.3.1.77)	1
	FNS	Flavone synthase	K13077 (1.14.11.22)	1
	FLS	Flavonol synthase	K05278 (1.14.11.23)	12
	C12RT1	Flavanone 7-O-glucoside 2^′′^-O-beta-L-rhamnosyltransferase	K12934 (2.3.1.172)	1
	FOMT	Flavonol 3-O-methyltransferase	K05279 (2.1.1.76)	9
Anthocyanin modification	GT1	Anthocyanidin 5′,3′-O-glucosyltransferase	K12938 (2.4.1.-)	7
	3′GT	Anthocyanin 3′-O-beta-glucosyltransferase	K12939 (2.4.1.238)	1
	5 MaT1	Anthocyanin 5-O-glucoside-6^′′′^-O-malonyltransferase	K12934 (2.3.1.172)	1
	UFGT	Anthocyanidin 3-O-glucosyltransferase	K12930 (2.4.1.115)	5
	CrOMT2	Myricetin O-methyltransferase	K13272 (2.1.1.149)	1
	LuOMT	Luteolin O-methyltransferase	\^*d*^ (2.1.1.75)	2

a *No. All, the total number of unigenes investigated*.

d *Omission of numbers for the KO id*.

To compare levels of pigment biosynthesis-related gene expression, relative expression levels of “petals vs. lips of purple-red *C. kanran*,” “petals vs. lips of white *C. kanran*,” “petals of white *C. kanran* vs. petals of purple-red *C. kanran*,” and “lips of white *C. kanran* vs. lips of purple-red *C. kanran*” were conducted. Among the differences in the gene expression profile of white *C. kanran* vs. purple-red *C. kanran*, each one of cinnamate 4-hydroxylase (C4H), CHS, DFR, and ANS were downregulated, whereas each one of F 3'H, flavonol synthase (FLS), and CCoAOMT were upregulated. In the same flower, two C4H and each one of CHS, DFR, and ANS were upregulated but only one CHS was downregulated (petals vs. lips of purple-red *C. kanran*), however, there was only one CHS downregulated (petals vs. lips of white *C. kanran*) (Table 2).

**Table 2:** Shifts in the expression levels of candidate genes related to flower pigmentation.

**Name**	**Target description**	**KO id (EC No.)**	**No. All^**a**^**	**No. Up^**b**^**	**No. Down^**c**^**
**Petals vs. lips of purple** ***C. kanran***					
C4H	Trans-cinnamate 4-monooxygenase	K00487 (1.14.13.11)	2	2	0
CHS	Chalcone synthase	K00660 (2.3.1.74)	2	1	1
DFR	Dihydroflavonol 4-reductase	K13082 (1.1.1.219)	1	1	0
ANS	Anthocyanidin synthase	K05277 (1.14.11.19)	1	1	0
**Petals of white vs. petals of purple** ***C. kanran***					
C4H	Trans-cinnamate 4-monooxygenase	K00487 (1.14.13.11)	2	0	1
CHS	Chalcone synthase	K00660 (2.3.1.74)	1	0	1
DFR	Dihydroflavonol 4-reductase	K13082 (1.1.1.219)	1	0	1
FLS	Flavonol synthase	K05278 (1.14.11.23)	12	1	0
ANS	Anthocyanidin synthase	K05277 (1.14.11.19)	1	0	1
CCoAOMT	Caffeoyl-CoAO-methyltransferase	K00588 (2.1.1.104)	1	1	0
**Petals vs. lips of white** ***C. kanran***					
CHS	Chalcone synthase	K00660 (2.3.1.74)	1	0	1
**Lips of white vs. lips of purple** ***C. kanran***					
F3'H	Flavonoid 3′-monooxygenase	K05280 (1.14.13.21)	1	1	0
FLS	Flavonol synthase	K05278 (1.14.11.23)	12	1	0

a *No. All, the total number of unigenes investigated*.

b *No. Up, the number of unigenes with higher expression level in the former than the latter*.

c *No. Down, the number of unigenes with lower expression level in the former than the latter*.

### Validating RNA-seq Data by qRT-PCR

**Figure 5:**
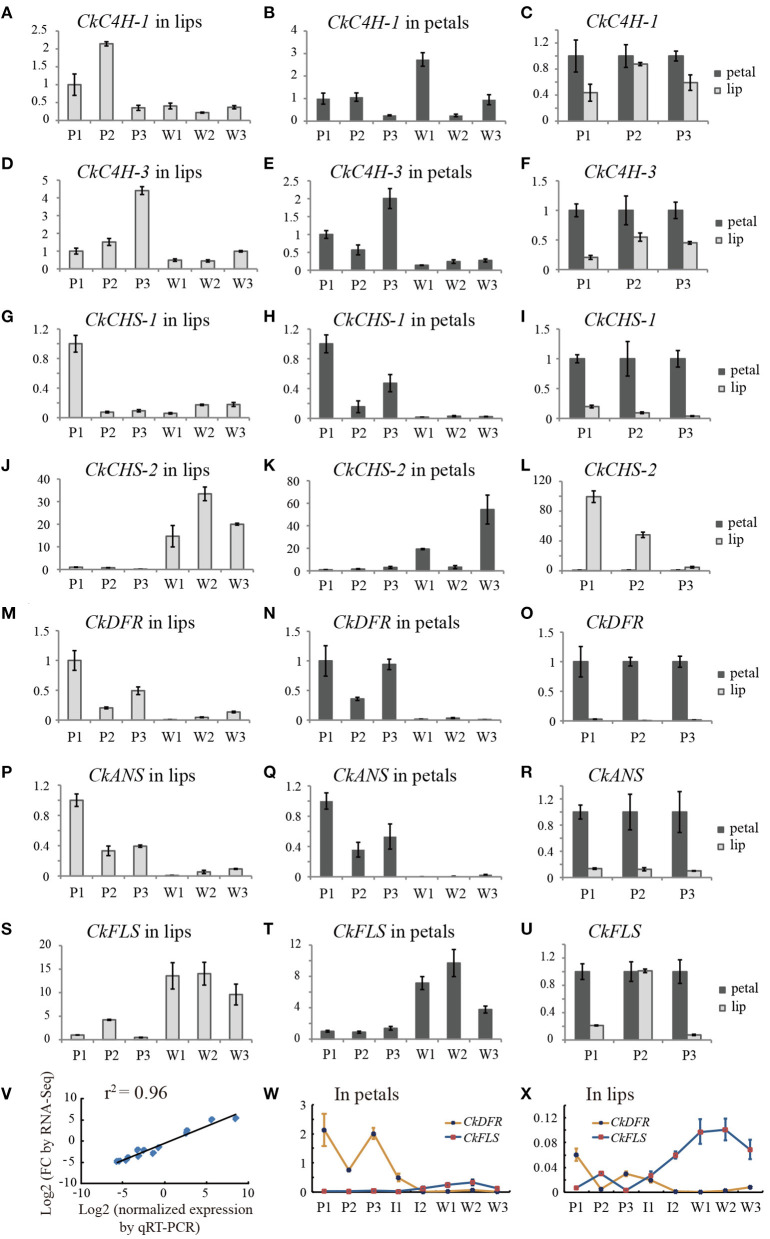
Transcript accumulation measurements of colour-related genes involved in the anthocyanidin metabolic process. **(A–U)** The qRT-PCR analysis of differentially expressed genes from RNA-Seq analysis. **(V)** Correlation of gene expression results obtained by qRT-PCR analysis and RNA-Seq for colour-related genes of the purple-red and white flower buds (length of approximately 3.0 cm). **(W,X)** line charts of the relative expression levels of *DFR* and *FLS* in petals and lips, respectively. The Y-axis of all charts except V represents the relative expression level. P1, purple-red *C. kanran* 1; W1, white *C. kanran* 1; I1, intermediate phenotype *C. kanran* 1.

To validate the RNA-seq expression profiles, seven differentially expressed unigenes encoding C4H, CHS, DFR, ANS, and FLS were quantified by qRT-PCR (Figures 5A–U). The general expression profile obtained by qRT-PCR was perfectly consistent with that obtained by RNA-seq (Figure 5V), supporting the accuracy of the RNA-seq data.

*CkC4H-3, CkCHS-1, CkDFR*, and *CkANS* were highly expressed in purple-red floral tissues and expressed at low levels in white floral tissues (Figures 5A–I, M–R). Conversely, *CkFLS* was expressed at a higher level both in petals and the lip of white *C. kanran* than purple-red *C. kanran* (Figures 5S,T). Most of the anthocyanin-related genes exhibited higher expression levels in petals than in the lip, but the opposite results appeared in the *CkCHS-2* expression pattern (Figures 5J–L). More interestingly, *CkCHS-2* was abundantly expressed in white flowers but expressed at low levels in purple-red flowers (Figures 5J,K). Conversely, *CkCHS-1* was highly expressed in purple-red flowers but had low expression levels in white flowers (Figures 5G,H), suggesting that white and purple-red flowers may have different *CHSs* to participate in the flavonoid/anthocyanin biosynthesis pathway. Additionally, *CkDFR* expression in three shades of purple-red flowers displayed a reverse trend to *CkFLS* (Figures 5W,X), which is in agreement with the competition to the same substrate between DFR and FLS.

### Pearson's Correlation Coefficient Between Gene Transcript Level and Flavonoid Content

**Figure 6:**
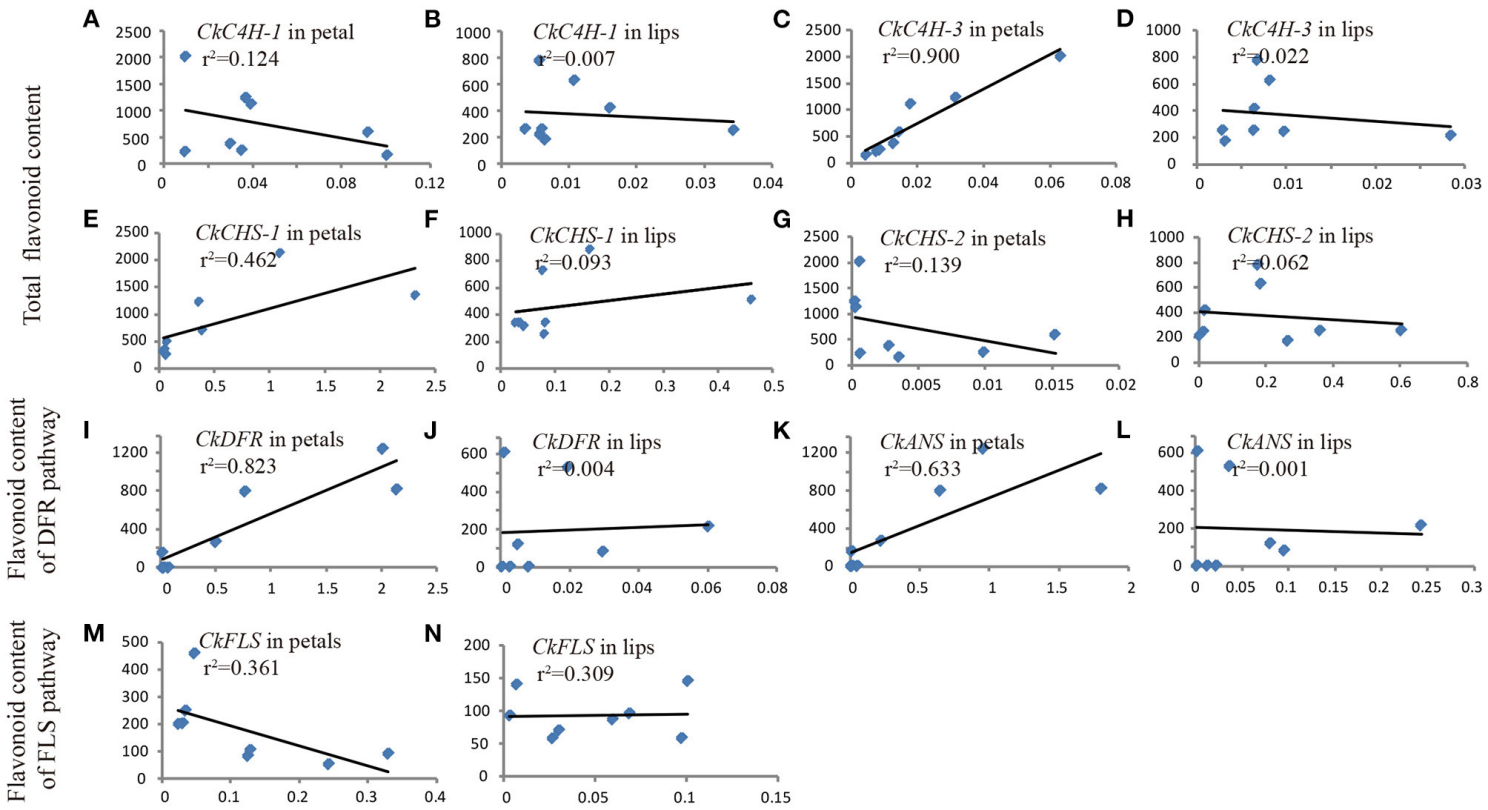
Pearson's correlation coefficient between gene transcript level (X-axis) and flavonoid content (Y-axis) in petals **(A,C,E,G,I,K,M)** and the lip **(B,D,F,H,J,L,N)** of *C. kanran*. Total flavonoids included MY, KA, QU, PEO, MA, and CY. The anthocyanidins of the DFR pathway included PEO, MA, and CY. The flavonol of the FLS pathway included MY, KA, and QU.

To uncover the relationship between gene expression and flavonoid content, the transcript abundance of flavonoid pathway genes and the accumulation of related products were detected in three shades of flowers (white, purple-red, and intermediate phenotype *C. kanran*). As presented in Figure 6, the concentration of flavonoids varied with the expression level of different genes. Normally, two variables show a strong correlation if the correlation coefficient (*r*) exceeds 0.6 in the Pearson correlation analysis. The contents of total flavonoids were significantly positively correlated with the expression levels of *CkC4H-3* and *CkCHS-1* in petals (*r*^2^ = 0.9055, *r*^2^ = 0.4642) (Figures 6C,E). However, there was poor or no correlation between the total flavonoid content and *CkC4H-1* and *CkCHS-2* transcripts (Figures 6AB,G,H), which agreed with the qRT-PCR results showing ruleless expression levels in purple-red and white *C. kanran* (Figures 6A–C,J–L). *CkDFR* and *CkANS* were involved in the biosynthesis of CY, MA, and PEO and were positively correlated with the anthocyanidin content of the DFR pathway in petals (Figures 6I,L).

### Production of Purple-Red Pigmentation in White *C. kanran* by Transient Expression of *CHS-1, DFR*, and *ANS* Genes

**Figure 7:**
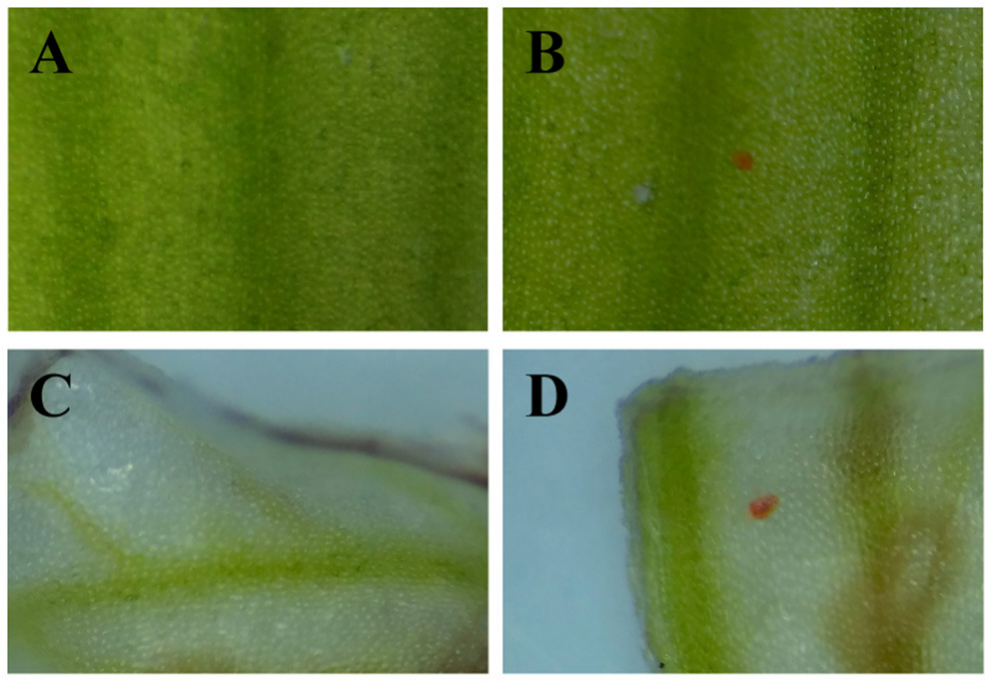
Development of red spots in white petals and lips due to transient expression of *CkCHS-1, CkDFR*, and *CkANS* under the control of the 35S promoter. **(A)** A petal conducted by particle bombardment with empty vector plasmid. **(B)** A petal conducted by particle bombardment with the mixture of overexpression vector plasmids of the three genes. **(C)** A lip conducted by particle bombardment with empty vector plasmid. **(D)** A lip conducted by particle bombardment with the mixture of overexpression vector plasmids of the three genes.

**Figure 8:**
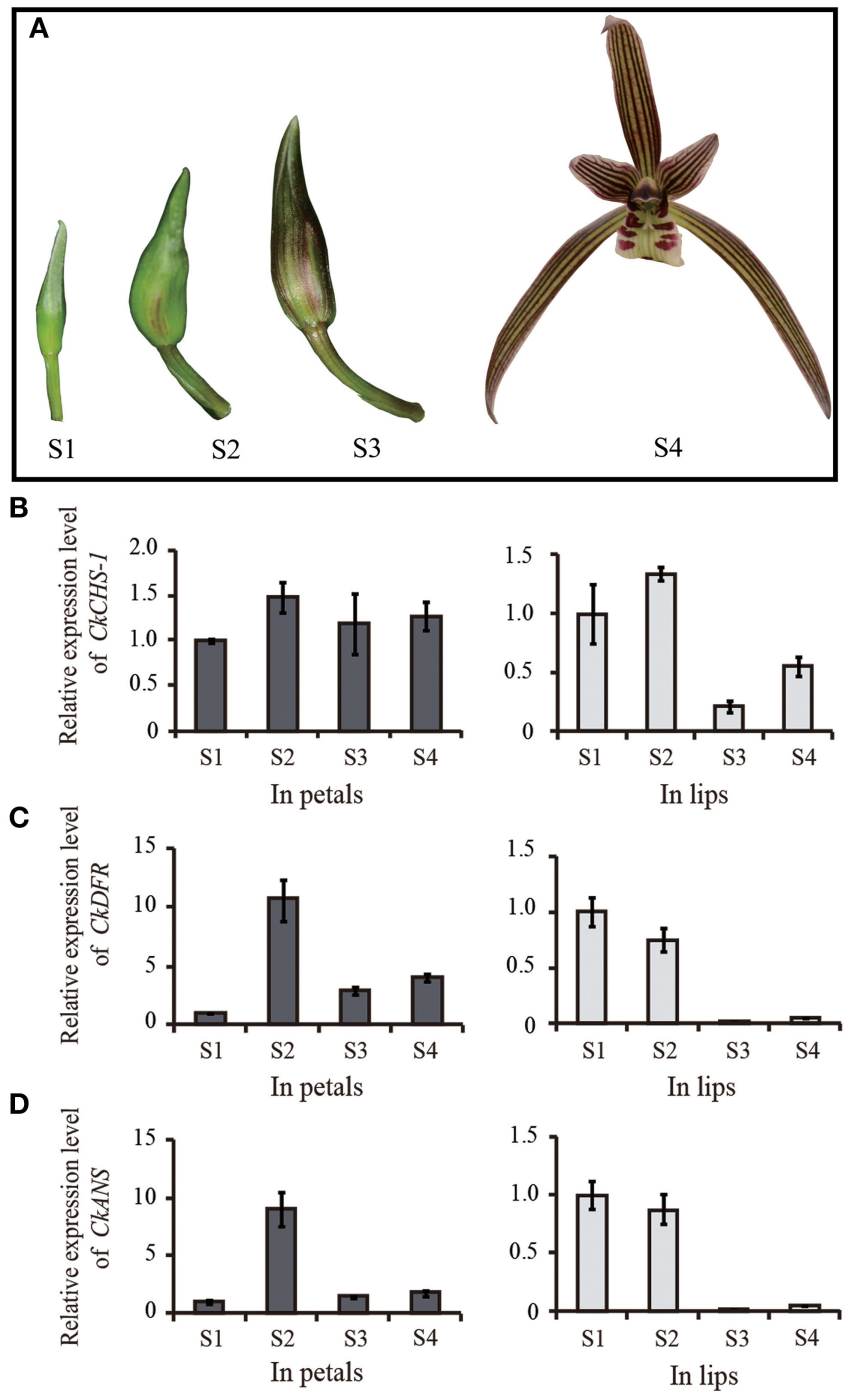
*CkCHS-1, CkDFR*, and *CkANS* expression levels were measured by qRT-PCR in four stages. **(A)** Four development stages of purple-red flower. **(B–D)** The relative expression level of *CkCHS-1, CkDFR*, and *CkANS* in four development stages of petals and lips of purple-red flower, respectively. Four developmental stages: stage 1 (S1, bud length of approximately 2 cm), stage 2 (S2, bud length of approximately 3 cm), stage 3 (S3, 1–2 days before anthesis), and stage 4 (S4, the flower opened).

According to Pearson's correlation analysis in Figure 6, we conjectured that the recovery of the *CkCHS-1, CkDFR*, and *CkANS* genes in the white type could complete the anthocyanin pathway to display a red pigment similar to that of purple-red *C. kanran*. Hence, these three genes were divided into three groups, which are single genes, double genes, and triple genes, to be validated for transient expression by particle bombardment. Then, the three groups of genes were bombarded into white petals and lips. After incubation on 1/2 MS medium for 5 days, only the group of triple genes accumulated red spots in both petal and lip tissue (Figure 7). This result supported that anthocyanin absence in white-type *C. kanran* probably resulted from the expression deficiency of *CkCHS-1, CkDFR*, and *CkANS*. We further evaluated the temporal transcription of these three genes in four developmental stages: stage 1 (S1, bud length of approximately 2 cm), stage 2 (S2, bud length of approximately 3 cm), stage 3 (S3, 1–2 days before anthesis), and stage 4 (S4, the flower opened) (Figure 8). In petals, the transcriptional profile showed that *CkCHS-1, CkDFR*, and *CkANS* were all actively expressed gradually from S1 to S4 and reached maximal expression in S2. Both *CkDFR* and *CkANS* presented a sharp peak at S2, but *CkCHS-1* maintained similar transcriptional activity during all stages in petals. These three gene expression patterns in the lip displayed a similar trend of higher expression in early stages S1 and S2, while *CkDFR* and *CkANS* showed the same trend in both organs.

## Discussion

The main pigments targeted for orchid flowers are anthocyanins that contribute to a variety of colours, such as red, purple, pink, and blue, while studies have shown that the main types of anthocyanidin aglycones in orchids are cyanidin, peonidin, and delphinidin (Liu et al., 2012; Vignolini et al., 2012; Wang et al., 2014; Liang et al., 2020; Zhang et al., 2020). In *Oncidium* Gower Ramsey, the red portion of lips contained a mixture of cyanidin-3-O-glucoside, delphinidin-3-O-glucoside, and peonidin-3-O-glucoside compounds (Liu et al., 2012). Flowers of *Phalaenopsis* with red-purple, purple, purple-violet, and violet to violet-blue colour were due to highly accumulated cyanidin-based anthocyanins, but no true-blue colour and no delphinidin was detected (Liang et al., 2020). In some cultivars of *Cymbidium hybrids*, only cyanidin-, malvidin-, and peonidin-type anthocyanins were identified (Woltering and Somhorst, 1990; Johnson et al., 1999). In *C. kanran*, the flavonoid composition exhibited a sharp shift between purple-red and white flowers. Three types (cyanidin, peonidin, and malvidin) of anthocyanidins were also detected in purple-red flowers but were absent in white flowers. While the transcript level of the colour-related gene showed that *CkC4H-3, CkCHS-1, CkDFR*, and *CkANS* were highly expressed corresponding to purple-red floral tissues compared with white type floral tissues, but *CkFLS* had a higher level of expression in the white one, and a similar result was also achieved in *Pleione limprichtii* (Zhang et al., 2020). Dihydroflavonol reductase is a key enzyme in the biosynthesis of anthocyanins and can catalyze the reduction of three colourless dihydroflavonols, DHQ, DHK, and DHM, to leucoanthocyanidins. Because of the similar structure of the three substrates, it is easy to understand that DFRs from many species can utilise all three substrates (Heller et al., 1985; Meyer et al., 1987; Stich et al., 1992; Helariutta et al., 1993; Tanaka et al., 1995; Livak and Schmittgen, 2001; Ma et al., 2008; Chen et al., 2013). dihydroflavonol reductases from some species specifically convert one or two types of dihydroflavonols. In *Petunia* and *Cymbidium*, pelargonidin-based brick red/orange flowers do not exist in nature because DFRs cloned from these two species cannot reduce DHK efficiently (Gerats et al., 1982; Forkmann and Ruhnau, 1987; Johnson et al., 1999), suggesting that DFRs have a striking substrate specificity (Springob et al., 2003). In *Fragaria* species, two DFRs were isolated, and one enzyme variant strongly preferred DHK as a substrate, whereas the other had a higher specificity for DHQ than for DHM (Miosic et al., 2014). Similar results were also reported in *Ginkgo biloba* and *Freesia hybrida* (Cheng et al., 2013; Li et al., 2017). In *C. kanran*, relative quantification showed that cyanidin, peonidin, and malvidin were detected, and the cyanidin content was far greater than that of peonidin and malvidin, indicating that the DFR isolated from *C. kanran* could catalyze DHQ conversion to leucocyanidin. However, more *in vitro* enzyme assays are needed to verify the substrate specificity of DFR with DHM and DHK.

Orchid flowers are striking for their specific patterns of colours in the perianths, and the regulation and formation of pigmentation is determined by specific cells of the different floral organs (Yu and Goh, 2001). In the case of *C. kanran*, the distributions of anthocyanin production depended on the tissue specificity. The pigmentation in the petals showed anthocyanins concentrated in the outer layer of subepidermal cells to the xylem (Figure 2A), but in the lip, the red anthocyanins only accumulated in epidermal cells (Figure 2C). Orchid flowers have three petals, and one of the petals is morphologically different in structure and is known as the lip or labellum. However, the differences between the petals and lip were represented not only in shape but also in tissue and genes. The epidermal cells in the lip displayed different forms, in which the adaxial side cells were large and bubble-like, but the abaxial side cells were similar to the petal epidermal cells, which were small and flat. However, anthocyanins accumulated in the region from subepidermal cells to the xylem in the petal (Figure 2A), while in the lip, anthocyanins accumulated only in the epidermal cells of the adaxial surface (Figure 2C). Accordingly, pigments in the adaxial side of lips displayed bright-coloured pigmentation compared with those in petals (Figures 1J,K, 2A,C). Similar results were also reported in the leaves of *Tipularia discolour* orchid, the same anthocyanins in different anatomical locations led to a broad range of colours (Hughes et al., 2020). In addition, differentially expressed genes in transcriptome analysis of *C. kanran* supported that the molecular changes also existed between the lip and petals. In petals, *CkCHS-1* was highly expressed, but *CkCHS-2* was inversely expressed in the lip. In the *Petunia hybrid*, 12 genes encode CHS from a gene family, which were detected specifically in the margin of the corolla throughout all stages of flower development. Among them, *CHS-A* and *CHS-J* were expressed predominantly. These results indicated that *CHS* expression is regulated by different mechanisms in marginal picotee petals (Saito et al., 2006). According to a previous study, MADS-box genes might collectively regulate the genes involved in colour differentiation in petals and sepals during *Cattleya* orchid flower development, which may result in the spatiotemporal expression of anthocyanin biosynthesis pathway structural genes to determine colour differentiation (Li et al., 2020).

Knowledge of the molecular genetic basis of the flavonoid biosynthetic pathway allows us to improve the colour quality of plants. The simplest strategy to modify flower colour is to change the amounts of endogenous anthocyanins by regulating the essential enzymes required for flavonoid biosynthesis. For example, transgenic gentian plants with a suppressed ANS showed pale-blue-coloured flowers, while those with a suppressed CHS exhibited a more serious phenotype, i.e., pale-blue to pure white flowers (Nakatsuka et al., 2008). The manipulation of DFR and FLS, which are competing steps for flux towards anthocyanins and flavonols, led to an increase or decrease in anthocyanin content in the flower (Davies and Schwinn, 2010). In addition, the approach of regulating endogenous modification genes has also been utilised in genetic engineering. For example, F3'5'H suppression by RNAi induced a flower colour change from purple to red in cyclamen (Boase et al., 2010). Similar transformations were also displayed in both F3'5'H and A5/3'AT (anthocyanin 5,3'-aromaticacyltransferase)-suppressed transgenic gentian plants (Nishihara and Nakatsuka, 2010). In *C. kanran*, the validation of the transient expression of key genes involved in colour formation showed that only triple genes, *CkCHS-1, CkDFR*, and *CkANS* could accumulate red spots in both the petal and lip tissue, indicating that the regulatory mechanism of coloration is complicated. To date, studies on some genetically defined genotypes or mutants revealing blocks of anthocyanin pathway gene expression have already been verified in many plants. In *Forsythia*, it was shown to be blocked in anthers at both the DFR and ANS levels but at the ANS level, only in petals (Rosati et al., 1999). However, the absence of anthocyanins in the skins of white berry varieties is due to a block only of UFGT expression in *Vitis* (Boss et al., 1996). Additionally, a point mutation of the CHS gene could result in the formation of the white flower of *Matthiola incana* (Hemleben et al., 2004). Another study in *Oncidium* also revealed that the failure of anthocyanin accumulation may be due to the inactivation of *OgCHS* caused by epigenetic methylation of the 5'-upstream promoter regions (Liu et al., 2012). However, the silencing of three different CHS genes of the purple-colour flower of *Dianthus chinensis via* VIGS could obtain white or pale purple flower (Liu et al., 2021), suggesting that several CHS genes may be involved in the formation of coloration. In *C. kanran*, it is noteworthy that *CkCHS-2* was abundantly expressed in white flowers but expressed at low levels in purple-red flowers (Figures 5J,K), and *CkCHS-1* was highly expressed in purple-red flowers but had lower expression levels in white flowers (Figures 5G,H), indicating the complicated regulation of CHSs in *C. kanran*. Therefore, further studies need to be performed on the regulatory mechanism of anthocyanin pathway-related genes in *C. kanran*.

**Figure 9:**
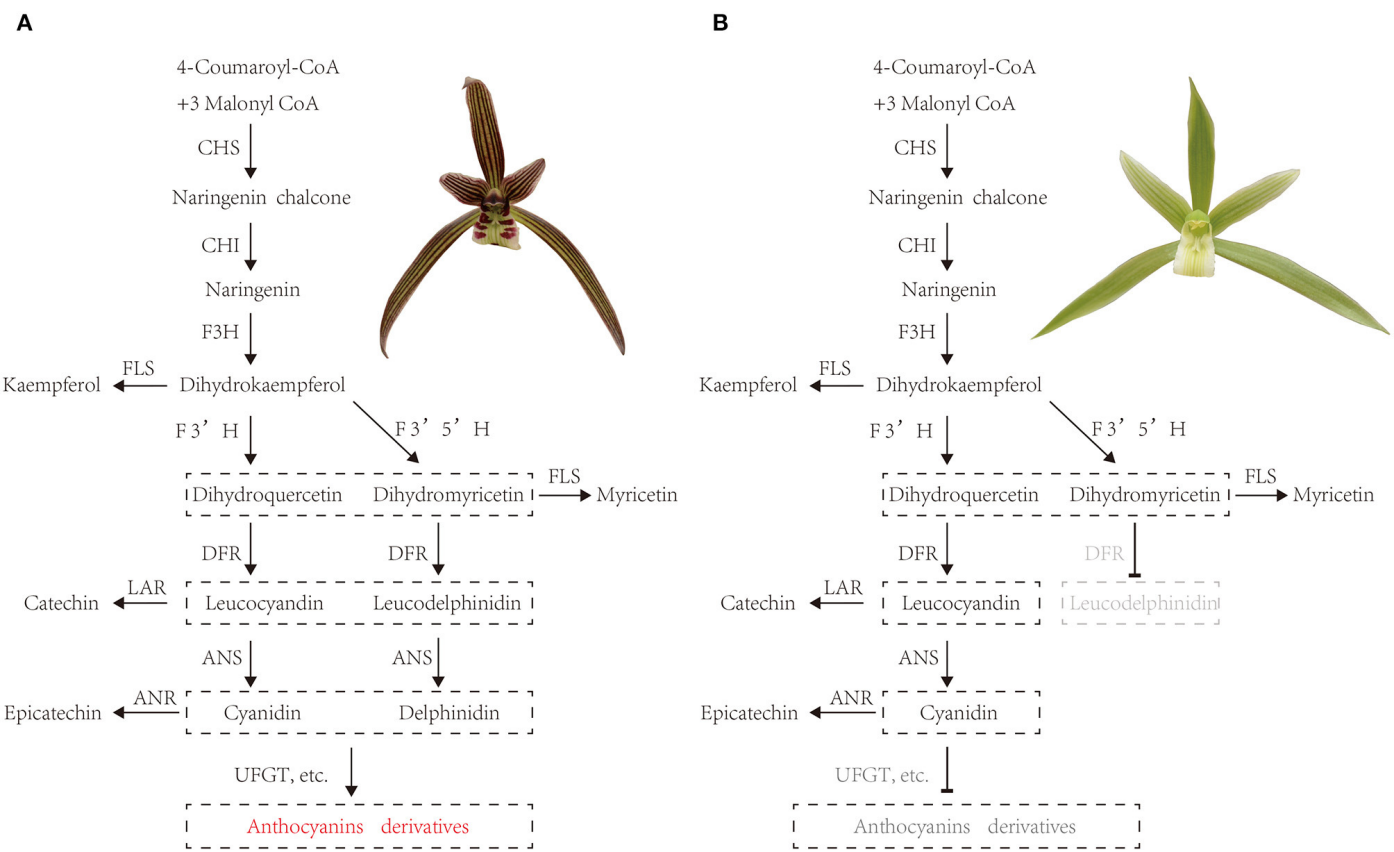
Tentative pathways for **(A)** purple-red and white **(B)** *C. kanran*. CHS, chalcone synthase; CHI, chalcone isomerase; F3H, flavanone 3-hydroxylase; F3'H, flavonoid 3'-hydroxylase; F3'5'H, flavonoid 3'5'-hydroxylase; DFR, dihydroflavonol 4-reductase; ANR, anthocyanidin reductase; ANS, anthocyanidin synthase; FLS, flavonol synthase; LAR, leucoanthocyanidin reductase; UFGT, anthocyanidin 3-O-glucosyltransferase.

Thus, we tentatively speculated about the pathways for purple-red and white *C. kanran* (Figure 9). In purple-red *C. kanran*, flavonoids are initially derived from phenylalanine and phenylpropanoid metabolism. Subsequently, *CkCHS* is generated to produce chalcones, and *CkCHI* catalyzes chalcones into naringenin. Furthermore, naringenin can be converted through *CkF3H, CkF3'H*, and *CkF3'5'H* to produce dihydroxyflavonols including DHK, DHQ, and DHM. Following the above reaction, *CkDFR* and *CkANS* further catalyze the divergent conversion of dihydroflavonols to produce colourless cyanidin and delphinidin. In the end, *CkUFGT* and other GTs catalyze the glucosylation of anthocyanidins (peonidin, malvidin, and cyanidin). As for white *C. kanran*, the main divergence of flavonoid metabolism is anthocyanin metabolism, the GTs cannot be regulated and fulfilled to catalyze the glucosylation of anthocyanidins. White-type flowers of *Cymbidium* are uncommon in nature, and traditional division propagation requires a long period, which leads to a low reproductive rate and high price. In this study, the molecular mechanism of anthocyanidin deficiency in white-type flowers establishes a foundation for genetic engineering to generate white-type *C. kanran*. The simplest strategy is to suppress the expression levels of *CkCHS-1, CkDFR*, and *CkANS*.

Concurrently, the regulatory mechanism of genes involved in the anthocyanin metabolic pathway has also been carried out in orchids. In *Oncidium, OgMYB1* directly activated the expression of *OgCHI* and *OgDFR* to regulate anthocyanin accumulation in the lip (Chiou and Yeh, 2008). In *Phalaenopsis*, three MYB transcription factors determine full-red pigmentation, red spots, and venation patterns (Hsu et al., 2015). A recent study in *C. hybrids* also indicated that *CyMYB1* regulates temporal- and temperature-dependent anthocyanin accumulation in petals (Nakatsuka et al., 2019). In *C. kanran*, we also isolated several differentially expressed *MYB* genes by Biochemical and Biophysical Research Communications transcriptome analysis and attempted to reveal the molecular mechanism of *MYB* genes regulating pigmentation patterning, which may provide genetic resources for *C. kanran* molecular breeding in the future.

## Conclusions

The white and purple-red phenotype *C. kanran* flowers had provided a unique experimental system to examine the mechanism for flower anthocyanin pigmentation. Compared with white flowers, purple-red flowers are comprised of three types of anthocyanidin compounds, namely, peonidin, malvidin, and cyanidin, which were shown by HPLC analysis. A total of 102 candidate genes involved in the flower coloration of *C. kanran* were identified using transcriptome analysis. Gene expression profiles showed that four of these genes were positively correlated with anthocyanidin biosynthesis by qRT-PCR. Transient expression of *CkCHS-1, CkDFR*, and *CkANS* by particle bombardment results in the accumulation of anthocyanins in white flowers. These results indicated that *CkCHS-1, CkDFR*, and *CkANS* are the key genes involved in floral pigment accumulation in *C. kanran*. This study also provided a platform for *C. kanran* functional genomic research.

## Data Availability Statement

The datasets presented in this study can be found in online repositories. The name of the repository and accession number can be found below: National Centre for Biotechnology Information (NCBI) BioProject, https://www.ncbi.nlm.nih.gov/bioproject/, PRJNA744371.

## Author Contributions

ZZ, XW, and ZL designed the experiments. ZZ, XW, YY, and ZY performed all experiments. ZZ, XW, and ZW analysed the data. ZZ and XW wrote the manuscript. ZL revised the manuscript. All the authors have read and agreed to the published version of the manuscript.

## Funding

This research was funded by the Natural Science Foundation of Zhejiang Province (grant nos. LY20C160005 and LY19C150003), National Key R & D Program of China (grant nos. 2018YFD1000401 and 2019YFD1000400), and Wenzhou Agricultural New Variety Breeding Cooperative Group Project (grant no. 2019ZX004-3).

## Conflict of Interest

The authors declare that the research was conducted in the absence of any commercial or financial relationships that could be construed as a potential conflict of interest.

## Publisher's Note

All claims expressed in this article are solely those of the authors and do not necessarily represent those of their affiliated organizations, or those of the publisher, the editors and the reviewers. Any product that may be evaluated in this article, or claim that may be made by its manufacturer, is not guaranteed or endorsed by the publisher.
